# Triaqua(2-{[(*E*)-5-formyl-2-oxidobenzyl­idene]amino}ethanesulfonato)cobalt(II) dihydrate

**DOI:** 10.1107/S1600536809031274

**Published:** 2009-08-12

**Authors:** Ju-Lan Zeng, Yi-Min Jiang, Li-Xian Sun, Zhong Cao, Dao-Wu Yang

**Affiliations:** a School of Chemistry and Biological Engineering, Changsha University of Science & Technology, Changsha 410004, People’s Republic of China; b College of Chemistry and Chemical Engineering, Guangxi Normal University, Guilin 541004, People’s Republic of China

## Abstract

The title compound, [Co(C_10_H_9_NO_5_S)(H_2_O)_3_]·2H_2_O, is a cobalt–Schiff base complex derived from taurine. There are two complex mol­ecules and four solvent water mol­ecules in the asymmetric unit. The central Co atom is six coordinated by two O atoms and one N atom of the ligand and three O atoms of water mol­ecules, forming a slightly distorted octa­hedral geometry. The crystal structure is stabilized by several O—H⋯O hydrogen bonds.

## Related literature

For general background, see Roth *et al.* (1993 ▶); Casella & Gullotti (1981 ▶, 1986 ▶); Wang *et al.* (1994 ▶). For related structures, see Zeng *et al.* (2003 ▶); Jiang *et al.* (2003 ▶); (2004 ▶); Zhang *et al.* (2004 ▶, 2005 ▶); Liu *et al.* (2005 ▶); Li *et al.* (2006 ▶, 2007 ▶); Qin *et al.* (2008 ▶).
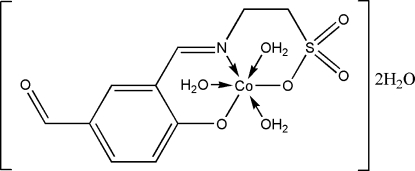


## Experimental

### 

#### Crystal data

[Co(C_10_H_9_NO_5_S)(H_2_O)_3_]·2H_2_O
*M* *_r_* = 404.25
Triclinic, 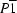

*a* = 7.748 (1) Å
*b* = 11.267 (3) Å
*c* = 18.941 (4) Å
α = 79.04 (2)°
β = 78.81 (1)°
γ = 89.59 (2)°
*V* = 1591.6 (6) Å^3^
*Z* = 4
Mo *K*α radiation
μ = 1.26 mm^−1^
*T* = 296 K
0.56 × 0.42 × 0.32 mm

#### Data collection

Siemens P4 diffractometer
Absorption correction: multi-scan (*SADABS*; Sheldrick, 1996 ▶) *T*_min_ = 0.852, *T*_max_ = 1.000 (expected range = 0.569–0.668)
6449 measured reflections
5764 independent reflections
4393 reflections with *I* > 2σ(*I*)
*R*_int_ = 0.014
3 standard reflections every 97 reflections intensity decay: 5.2%

#### Refinement

*R*[*F* ^2^ > 2σ(*F* ^2^)] = 0.038
*wR*(*F* ^2^) = 0.115
*S* = 1.06
5764 reflections
464 parameters
12 restraints
H atoms treated by a mixture of independent and constrained refinement
Δρ_max_ = 0.85 e Å^−3^
Δρ_min_ = −0.56 e Å^−3^

### 

Data collection: *XSCANS* (Siemens, 1994 ▶); cell refinement: *XSCANS*; data reduction: *SHELXTL* (Sheldrick, 2008 ▶); program(s) used to solve structure: *SHELXS97* (Sheldrick, 2008 ▶); program(s) used to refine structure: *SHELXL97* (Sheldrick, 2008 ▶); molecular graphics: *SHELXTL*; software used to prepare material for publication: *publCIF* (Westrip, 2009 ▶).

## Supplementary Material

Crystal structure: contains datablocks I, global. DOI: 10.1107/S1600536809031274/bt5026sup1.cif

Structure factors: contains datablocks I. DOI: 10.1107/S1600536809031274/bt5026Isup2.hkl

Additional supplementary materials:  crystallographic information; 3D view; checkCIF report

## Figures and Tables

**Table 1:** Hydrogen-bond geometry (Å, °)

*D*—H⋯*A*	*D*—H	H⋯*A*	*D*⋯*A*	*D*—H⋯*A*
O6—H6*OA*⋯O2^i^	0.825 (10)	1.954 (14)	2.772 (4)	171 (5)
O6—H6*OB*⋯O9^ii^	0.820 (10)	1.945 (18)	2.739 (3)	163 (5)
O7—H7*OA*⋯O13	0.816 (10)	2.45 (3)	3.194 (6)	153 (5)
O7—H7*OB*⋯O20	0.817 (10)	2.12 (3)	2.877 (9)	155 (7)
O8—H8*OA*⋯O17	0.827 (10)	1.924 (14)	2.742 (5)	170 (4)
O8—H8*OB*⋯O5^iii^	0.816 (10)	1.996 (13)	2.807 (4)	173 (5)
O14—H14*A*⋯O1	0.818 (10)	1.938 (17)	2.732 (3)	163 (5)
O14—H14*B*⋯O10^iv^	0.819 (10)	1.968 (12)	2.784 (4)	175 (5)
O15—H15*B*⋯O5^iii^	0.815 (10)	2.28 (4)	3.012 (5)	150 (7)
O15—H15*A*⋯O19	0.818 (10)	2.17 (5)	2.831 (7)	138 (7)
O16—H16*A*⋯O18	0.818 (10)	1.875 (12)	2.691 (5)	175 (4)
O16—H16*B*⋯O13^iii^	0.817 (10)	1.945 (17)	2.748 (5)	167 (6)

Symmetry codes: (i) 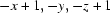; (ii) 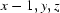; (iii) 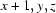; (iv) 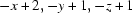.

**Fig. 1.**
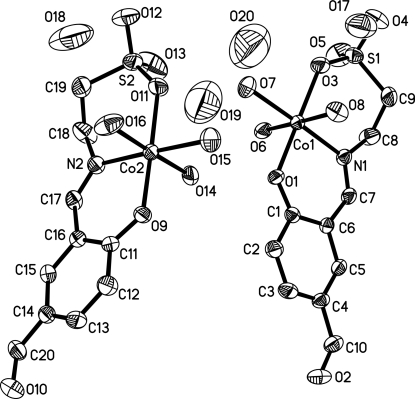
View of the asymmetric unit of the title compound, with displacement ellipsoids drawn at the 50% probability level. H atoms have been omitted for clarity.

**Fig. 2.**
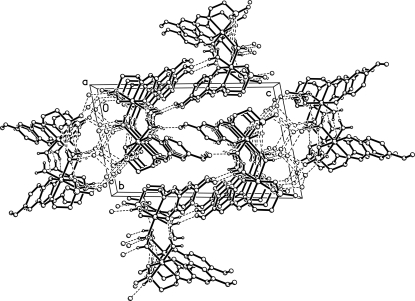
Packing diagram of the title compound along thea axis. H atoms except those involved in hydrogen-bond interactions have been omitted for clarity.

## supplementary crystallographic information

### Comment

In the title compound, there are two complex molecules and four solvent water molecules in the asymetric unit (Fig. 1). The central Co^II^ of the title compound is six coordinated with two O atoms, one N atom from the Schiff base ligand and three O atoms from the coordinated water, forming an slightly distorted octahedron structure. The Co atom forms two six-membered chelating rings with the ligand.   The bond lengths and angles involving Co1 and Co2   show minor differences  between the two complex molecules in the asymetric unit. The coordinating environment of central Co atom is similar to that reported by Qin *et al.* (2008). However, in the title compound, the bond length of the central Co and the O of sulfonate group (Co(1)—O(3) 2.085 (3) Å, Co(2)—O(11) 2.096 (3) Å) are shorter than that of central Co and O of coordinated water, and are even shorter than that of central Co and N of imine group. It indicates that the coordinating capability of the O atom of sulfonate group is stronger than that of water and N of imine group, derived from the two conjugated chelating rings and the electron-withdraw group of formyl on the benzene ring. With O—H···O hydrogen bonds, a three-dimensional network is formed as shown in Fig. 2.

### Experimental

The synthesis of the potassium salt of the Schiff base ligand 2-{[(*E*)-(2-hydroxy-5{[(2- sulfoethyl)imino]methyl}phenyl)methylidene]amino}-1- ethanesulfonic acid was reported previously (Zeng *et al.*, 2003). 1.0 mmol of ligand was dissolved in 15 ml of water. To this solution, 1.0 mmol of CoAc_2_.4H_2_O was added dropwise within 30 minutes while the mixture was stirred and heated at 50 °C. The mixture was stirred and heated at 50 °C for another 6 h, then cooled to room temperature. After filtration, the filtrate was left to stand at room temperature. Dark-red crystals suitable for X-raydiffraction were isolated several days later by natural evaporation, washed with methanol and dried in air. Yield 36%. Found(%): C, 29.03; H, 4.86; N,3.96, C_10_H_19_CoNO_10_S. Calcd.(%): C, 29.71; H, 4.70%; N,3.47%. IR: 1042.3, 1147.1, 1185.1, 1216.3(*υ -*SO_3_^-^); 1629.5(*υ*_C=N_); 3407.9(*υ*_O—H_).

### Refinement

The H atoms of the solvent water molecules could not be located from a difference Fourier map and were omitted from refinement. The H atoms of coordinated water were located in a difference Fourier map and their positions and isotropic displacement parameters were refined, with O—H restrainted   to O—H   0.82 (1) Å. All other H atoms were positioned geometrically, treated as riding atoms and refined isotropically, with C—H distances of 0.93–0.97 Å and with *U*_iso_(H) =1.2Ueq(C).

### Crystal data

**Table:**

[Co(C_10_H_9_NO_5_S)(H_2_O)_3_]·2H_2_O	*Z* = 4
*M**_r_* = 404.25	*F*(000) = 836
Triclinic, *P*1	*D*_x_ = 1.687 Mg m^−^^3^
*a* = 7.748 (1) Å	Mo *K*α radiation, λ = 0.71073 Å
*b* = 11.267 (3) Å	Cell parameters from 28 reflections
*c* = 18.941 (4) Å	θ = 5.6–15.2°
α = 79.04 (2)°	µ = 1.26 mm^−^^1^
β = 78.81 (1)°	*T* = 296 K
γ = 89.59 (2)°	Block, red
*V* = 1591.6 (6) Å^3^	0.56 × 0.42 × 0.32 mm

### Data collection

**Table:**

Siemens P4 diffractometer	4393 reflections with *I* > 2σ(*I*)
Radiation source: normal-focus sealed tube	*R*_int_ = 0.014
graphite	θ_max_ = 25.3°, θ_min_ = 1.1°
ω scans	*h* = 0→9
Absorption correction: multi-scan (*SADABS*; Sheldrick, 1996)	*k* = −13→13
*T*_min_ = 0.852, *T*_max_ = 1.000	*l* = −22→22
6449 measured reflections	3 standard reflections every 97 reflections
5764 independent reflections	intensity decay: 5.2%

### Refinement

**Table:**

Refinement on *F*^2^	Secondary atom site location: difference Fourier map
Least-squares matrix: full	Hydrogen site location: inferred from neighbouring sites
*R*[*F*^2^ > 2σ(*F*^2^)] = 0.038	H atoms treated by a mixture of independent and constrained refinement
*wR*(*F*^2^) = 0.115	*w* = 1/[σ^2^(*F*_o_^2^) + (0.072*P*)^2^] where *P* = (*F*_o_^2^ + 2*F*_c_^2^)/3
*S* = 1.06	(Δ/σ)_max_ < 0.001
5764 reflections	Δρ_max_ = 0.85 e Å^−^^3^
464 parameters	Δρ_min_ = −0.56 e Å^−^^3^
12 restraints	Extinction correction: *SHELXL*, Fc^*^=kFc[1+0.001xFc^2^λ^3^/sin(2θ)]^-1/4^
Primary atom site location: structure-invariant direct methods	Extinction coefficient: 0.0031 (8)

### Special details

**Table:**

Geometry. All e.s.d.'s (except the e.s.d. in the dihedral angle between two l.s. planes) are estimated using the full covariance matrix. The cell e.s.d.'s are taken into account individually in the estimation of e.s.d.'s in distances, angles and torsion angles; correlations between e.s.d.'s in cell parameters are only used when they are defined by crystal symmetry. An approximate (isotropic) treatment of cell e.s.d.'s is used for estimating e.s.d.'s involving l.s. planes.
Refinement. Refinement of *F*^2^ against ALL reflections. The weighted *R*-factor *wR* and goodness of fit *S* are based on *F*^2^, conventional *R*-factors *R* are based on *F*, with *F* set to zero for negative *F*^2^. The threshold expression of *F*^2^ > σ(*F*^2^) is used only for calculating *R*-factors(gt) *etc*. and is not relevant to the choice of reflections for refinement. *R*-factors based on *F*^2^ are statistically about twice as large as those based on *F*, and *R*- factors based on ALL data will be even larger.

### Fractional atomic coordinates and isotropic or equivalent isotropic displacement parameters (Å^2^)

**Table:**

	*x*	*y*	*z*	*U*_iso_*/*U*_eq_	
Co1	0.39588 (6)	0.16129 (4)	0.22274 (2)	0.02522 (14)	
S1	0.10792 (13)	0.07289 (9)	0.13827 (5)	0.0355 (2)	
O1	0.5728 (3)	0.1640 (2)	0.28894 (13)	0.0309 (6)	
O2	0.8277 (4)	−0.1552 (3)	0.56195 (14)	0.0439 (7)	
O3	0.2421 (4)	0.1591 (2)	0.14400 (14)	0.0408 (6)	
O4	0.1212 (5)	0.0544 (3)	0.06499 (18)	0.0694 (10)	
O5	−0.0634 (4)	0.1062 (3)	0.1714 (2)	0.0662 (10)	
O6	0.1953 (4)	0.2399 (2)	0.28909 (15)	0.0388 (6)	
O7	0.4814 (5)	0.3345 (3)	0.16357 (19)	0.0521 (8)	
O8	0.5882 (4)	0.0785 (3)	0.15431 (16)	0.0397 (6)	
N1	0.2960 (4)	−0.0072 (2)	0.28662 (16)	0.0275 (6)	
C1	0.6084 (4)	0.0813 (3)	0.34091 (18)	0.0245 (7)	
C2	0.7575 (5)	0.0979 (3)	0.3726 (2)	0.0329 (8)	
H2	0.8324	0.1650	0.3524	0.039*	
C3	0.7932 (5)	0.0190 (3)	0.4310 (2)	0.0334 (8)	
H3	0.8917	0.0325	0.4501	0.040*	
C4	0.6816 (5)	−0.0842 (3)	0.46352 (18)	0.0294 (8)	
C5	0.5419 (5)	−0.1048 (3)	0.43156 (19)	0.0281 (7)	
H5	0.4703	−0.1735	0.4517	0.034*	
C6	0.5031 (4)	−0.0269 (3)	0.37025 (18)	0.0254 (7)	
C7	0.3537 (5)	−0.0620 (3)	0.34235 (19)	0.0284 (7)	
H7	0.2928	−0.1328	0.3681	0.034*	
C8	0.1392 (5)	−0.0632 (4)	0.2712 (2)	0.0441 (10)	
H8A	0.0365	−0.0179	0.2869	0.053*	
H8B	0.1215	−0.1450	0.2995	0.053*	
C9	0.1554 (6)	−0.0669 (3)	0.1902 (2)	0.0454 (10)	
H9A	0.0752	−0.1291	0.1853	0.055*	
H9B	0.2741	−0.0885	0.1707	0.055*	
C10	0.7083 (5)	−0.1653 (3)	0.5296 (2)	0.0356 (9)	
H10	0.6285	−0.2300	0.5487	0.043*	
Co2	0.88816 (6)	0.51076 (4)	0.22408 (2)	0.02608 (15)	
S2	0.59985 (13)	0.65643 (9)	0.13838 (5)	0.0374 (2)	
O9	1.0676 (3)	0.4671 (2)	0.28928 (13)	0.0310 (5)	
O10	1.3260 (4)	0.6176 (3)	0.55918 (15)	0.0452 (7)	
O11	0.7245 (4)	0.5601 (2)	0.14791 (16)	0.0449 (7)	
O12	0.6213 (5)	0.7151 (3)	0.06321 (18)	0.0702 (10)	
O13	0.4233 (4)	0.6130 (4)	0.1716 (2)	0.0824 (12)	
O14	0.6910 (4)	0.3906 (2)	0.29177 (15)	0.0387 (6)	
O15	0.9771 (5)	0.3737 (3)	0.1658 (2)	0.0527 (8)	
O16	1.0798 (4)	0.6313 (3)	0.15392 (18)	0.0515 (8)	
N2	0.7908 (4)	0.6426 (3)	0.28574 (16)	0.0307 (7)	
C11	1.1037 (4)	0.5178 (3)	0.34031 (18)	0.0259 (7)	
C12	1.2544 (5)	0.4818 (3)	0.3713 (2)	0.0348 (8)	
H12	1.3302	0.4275	0.3510	0.042*	
C13	1.2905 (5)	0.5245 (3)	0.4294 (2)	0.0344 (8)	
H13	1.3897	0.4987	0.4481	0.041*	
C14	1.1794 (5)	0.6072 (3)	0.46160 (19)	0.0302 (8)	
C15	1.0381 (5)	0.6493 (3)	0.42994 (19)	0.0282 (7)	
H15	0.9669	0.7065	0.4497	0.034*	
C16	0.9990 (5)	0.6087 (3)	0.36925 (18)	0.0268 (7)	
C17	0.8501 (5)	0.6630 (3)	0.34058 (19)	0.0301 (8)	
H17	0.7906	0.7196	0.3653	0.036*	
C18	0.6368 (6)	0.7123 (4)	0.2686 (2)	0.0497 (11)	
H18A	0.6216	0.7781	0.2955	0.060*	
H18B	0.5320	0.6601	0.2848	0.060*	
C19	0.6555 (6)	0.7637 (3)	0.1876 (3)	0.0486 (11)	
H19A	0.7761	0.7924	0.1677	0.058*	
H19B	0.5801	0.8324	0.1812	0.058*	
C20	1.2063 (5)	0.6470 (3)	0.5267 (2)	0.0347 (8)	
H20	1.1252	0.6997	0.5456	0.042*	
O17	0.5837 (6)	0.1098 (4)	0.0076 (2)	0.0804 (12)	
O18	1.0564 (7)	0.8005 (5)	0.0353 (3)	0.126 (2)	
O19	1.2760 (9)	0.4225 (5)	0.0508 (3)	0.140 (2)	
O20	0.7769 (13)	0.3685 (6)	0.0427 (4)	0.192 (3)	
H6OA	0.186 (7)	0.222 (4)	0.3340 (7)	0.066 (16)*	
H6OB	0.161 (6)	0.3091 (19)	0.280 (3)	0.065 (16)*	
H7OA	0.498 (7)	0.402 (2)	0.172 (3)	0.074 (17)*	
H7OB	0.543 (8)	0.336 (6)	0.1231 (18)	0.11 (3)*	
H8OA	0.579 (6)	0.095 (4)	0.1108 (9)	0.047 (13)*	
H8OB	0.687 (3)	0.092 (4)	0.160 (3)	0.063 (16)*	
H14B	0.680 (6)	0.385 (4)	0.3362 (7)	0.050 (14)*	
H14A	0.671 (6)	0.324 (2)	0.283 (3)	0.062 (15)*	
H15A	1.019 (10)	0.384 (7)	0.1220 (11)	0.14 (3)*	
H15B	0.967 (10)	0.3018 (17)	0.184 (4)	0.12 (3)*	
H16A	1.068 (5)	0.680 (3)	0.1173 (14)	0.041 (12)*	
H16B	1.184 (2)	0.637 (5)	0.156 (3)	0.09 (2)*	

### Atomic displacement parameters (Å^2^)

**Table:**

	*U*^11^	*U*^22^	*U*^33^	*U*^12^	*U*^13^	*U*^23^
Co1	0.0288 (3)	0.0215 (2)	0.0267 (3)	−0.00002 (18)	−0.0105 (2)	−0.00297 (17)
S1	0.0346 (5)	0.0398 (5)	0.0376 (5)	0.0035 (4)	−0.0193 (4)	−0.0092 (4)
O1	0.0386 (14)	0.0232 (12)	0.0320 (13)	−0.0050 (10)	−0.0168 (11)	0.0026 (10)
O2	0.0445 (17)	0.0550 (17)	0.0336 (15)	0.0099 (13)	−0.0186 (13)	−0.0013 (12)
O3	0.0467 (17)	0.0376 (14)	0.0424 (16)	−0.0020 (12)	−0.0247 (13)	−0.0019 (12)
O4	0.102 (3)	0.073 (2)	0.0461 (19)	−0.001 (2)	−0.0377 (19)	−0.0213 (17)
O5	0.0338 (17)	0.080 (2)	0.083 (2)	0.0136 (16)	−0.0197 (17)	−0.0041 (19)
O6	0.0530 (18)	0.0322 (14)	0.0327 (16)	0.0157 (13)	−0.0122 (13)	−0.0070 (12)
O7	0.074 (2)	0.0296 (15)	0.050 (2)	−0.0173 (15)	−0.0160 (18)	0.0040 (14)
O8	0.0302 (16)	0.0518 (17)	0.0382 (17)	0.0038 (13)	−0.0062 (13)	−0.0121 (13)
N1	0.0258 (15)	0.0237 (14)	0.0331 (16)	−0.0022 (12)	−0.0084 (13)	−0.0028 (12)
C1	0.0236 (17)	0.0261 (16)	0.0251 (17)	0.0031 (13)	−0.0051 (14)	−0.0078 (13)
C2	0.033 (2)	0.0312 (18)	0.035 (2)	−0.0053 (15)	−0.0119 (16)	−0.0019 (15)
C3	0.0287 (19)	0.041 (2)	0.034 (2)	0.0033 (16)	−0.0141 (16)	−0.0077 (16)
C4	0.035 (2)	0.0299 (18)	0.0236 (17)	0.0123 (15)	−0.0067 (15)	−0.0055 (14)
C5	0.0311 (19)	0.0225 (16)	0.0301 (18)	0.0045 (14)	−0.0059 (15)	−0.0036 (13)
C6	0.0284 (18)	0.0221 (16)	0.0266 (17)	0.0048 (13)	−0.0061 (14)	−0.0061 (13)
C7	0.0300 (19)	0.0219 (16)	0.0320 (19)	−0.0037 (14)	−0.0068 (15)	−0.0011 (14)
C8	0.040 (2)	0.042 (2)	0.050 (2)	−0.0151 (18)	−0.0230 (19)	0.0081 (18)
C9	0.055 (3)	0.031 (2)	0.058 (3)	−0.0027 (18)	−0.028 (2)	−0.0123 (18)
C10	0.040 (2)	0.035 (2)	0.0300 (19)	0.0119 (16)	−0.0066 (17)	−0.0036 (15)
Co2	0.0295 (3)	0.0223 (2)	0.0292 (3)	0.00488 (18)	−0.0111 (2)	−0.00628 (18)
S2	0.0355 (5)	0.0378 (5)	0.0421 (6)	0.0040 (4)	−0.0206 (4)	−0.0029 (4)
O9	0.0381 (14)	0.0282 (12)	0.0332 (13)	0.0124 (10)	−0.0173 (11)	−0.0121 (10)
O10	0.0445 (17)	0.0576 (17)	0.0392 (16)	−0.0009 (14)	−0.0172 (13)	−0.0139 (13)
O11	0.0575 (18)	0.0378 (14)	0.0520 (17)	0.0138 (13)	−0.0363 (15)	−0.0139 (12)
O12	0.104 (3)	0.061 (2)	0.0470 (19)	0.014 (2)	−0.0361 (19)	0.0065 (15)
O13	0.0363 (19)	0.136 (4)	0.082 (3)	−0.013 (2)	−0.0181 (18)	−0.030 (2)
O14	0.0518 (18)	0.0307 (14)	0.0344 (16)	−0.0106 (12)	−0.0103 (13)	−0.0055 (12)
O15	0.073 (2)	0.0399 (17)	0.052 (2)	0.0180 (16)	−0.0163 (18)	−0.0216 (15)
O16	0.0320 (17)	0.060 (2)	0.0513 (19)	−0.0042 (14)	−0.0085 (15)	0.0185 (15)
N2	0.0296 (16)	0.0291 (15)	0.0367 (17)	0.0083 (12)	−0.0108 (13)	−0.0106 (13)
C11	0.0283 (18)	0.0254 (16)	0.0243 (17)	0.0019 (14)	−0.0081 (14)	−0.0022 (13)
C12	0.035 (2)	0.037 (2)	0.036 (2)	0.0121 (16)	−0.0098 (17)	−0.0139 (16)
C13	0.032 (2)	0.037 (2)	0.038 (2)	0.0029 (16)	−0.0157 (16)	−0.0050 (16)
C14	0.036 (2)	0.0279 (17)	0.0259 (18)	−0.0054 (15)	−0.0064 (15)	−0.0029 (14)
C15	0.0291 (19)	0.0262 (17)	0.0302 (18)	−0.0003 (14)	−0.0049 (15)	−0.0086 (14)
C16	0.0293 (18)	0.0252 (16)	0.0260 (18)	0.0010 (14)	−0.0062 (14)	−0.0044 (13)
C17	0.0255 (18)	0.0296 (18)	0.039 (2)	0.0072 (14)	−0.0073 (16)	−0.0150 (15)
C18	0.039 (2)	0.067 (3)	0.061 (3)	0.030 (2)	−0.027 (2)	−0.037 (2)
C19	0.055 (3)	0.033 (2)	0.068 (3)	0.0159 (19)	−0.035 (2)	−0.013 (2)
C20	0.035 (2)	0.038 (2)	0.034 (2)	−0.0058 (16)	−0.0091 (17)	−0.0103 (16)
O17	0.095 (3)	0.096 (3)	0.055 (2)	0.032 (2)	−0.021 (2)	−0.020 (2)
O18	0.126 (4)	0.135 (4)	0.101 (4)	−0.010 (3)	−0.055 (3)	0.049 (3)
O19	0.211 (7)	0.095 (4)	0.095 (4)	0.009 (4)	0.012 (4)	−0.016 (3)
O20	0.281 (10)	0.131 (5)	0.141 (6)	0.019 (6)	0.005 (6)	−0.014 (4)

### Geometric parameters (Å, °)

**Table:**

Co1—O1	2.033 (2)	Co2—O9	2.031 (2)
Co1—O3	2.085 (3)	Co2—O11	2.096 (3)
Co1—O7	2.098 (3)	Co2—O16	2.096 (3)
Co1—O6	2.103 (3)	Co2—O15	2.101 (3)
Co1—N1	2.110 (3)	Co2—O14	2.102 (3)
Co1—O8	2.112 (3)	Co2—N2	2.108 (3)
S1—O4	1.426 (3)	S2—O12	1.430 (3)
S1—O5	1.434 (3)	S2—O13	1.437 (4)
S1—O3	1.461 (3)	S2—O11	1.457 (3)
S1—C9	1.768 (4)	S2—C19	1.763 (4)
O1—C1	1.291 (4)	O9—C11	1.286 (4)
O2—C10	1.222 (5)	O10—C20	1.220 (5)
O6—H6OA	0.825 (10)	O14—H14B	0.819 (10)
O6—H6OB	0.820 (10)	O14—H14A	0.818 (10)
O7—H7OA	0.816 (10)	O15—H15A	0.818 (10)
O7—H7OB	0.817 (10)	O15—H15B	0.815 (10)
O8—H8OA	0.827 (10)	O16—H16A	0.818 (10)
O8—H8OB	0.816 (10)	O16—H16B	0.817 (10)
N1—C7	1.276 (4)	N2—C17	1.273 (4)
N1—C8	1.476 (4)	N2—C18	1.478 (5)
C1—C6	1.429 (5)	C11—C16	1.427 (5)
C1—C2	1.429 (5)	C11—C12	1.428 (5)
C2—C3	1.354 (5)	C12—C13	1.357 (5)
C2—H2	0.9300	C12—H12	0.9300
C3—C4	1.419 (5)	C13—C14	1.409 (5)
C3—H3	0.9300	C13—H13	0.9300
C4—C5	1.380 (5)	C14—C15	1.387 (5)
C4—C10	1.452 (5)	C14—C20	1.441 (5)
C5—C6	1.397 (5)	C15—C16	1.400 (5)
C5—H5	0.9300	C15—H15	0.9300
C6—C7	1.449 (5)	C16—C17	1.452 (5)
C7—H7	0.9300	C17—H17	0.9300
C8—C9	1.523 (6)	C18—C19	1.512 (6)
C8—H8A	0.9700	C18—H18A	0.9700
C8—H8B	0.9700	C18—H18B	0.9700
C9—H9A	0.9700	C19—H19A	0.9700
C9—H9B	0.9700	C19—H19B	0.9700
C10—H10	0.9300	C20—H20	0.9300
			
O1—Co1—O3	172.70 (11)	O9—Co2—O11	174.22 (11)
O1—Co1—O7	91.29 (11)	O9—Co2—O16	87.25 (12)
O3—Co1—O7	85.59 (12)	O11—Co2—O16	87.63 (12)
O1—Co1—O6	94.61 (11)	O9—Co2—O15	90.74 (12)
O3—Co1—O6	91.96 (11)	O11—Co2—O15	86.58 (12)
O7—Co1—O6	89.35 (14)	O16—Co2—O15	90.10 (15)
O1—Co1—N1	89.11 (10)	O9—Co2—O14	94.59 (11)
O3—Co1—N1	94.45 (11)	O11—Co2—O14	90.52 (12)
O7—Co1—N1	176.05 (14)	O16—Co2—O14	178.13 (12)
O6—Co1—N1	86.70 (11)	O15—Co2—O14	89.50 (14)
O1—Co1—O8	87.77 (11)	O9—Co2—N2	89.28 (10)
O3—Co1—O8	85.74 (11)	O11—Co2—N2	93.63 (11)
O7—Co1—O8	91.78 (14)	O16—Co2—N2	92.44 (13)
O6—Co1—O8	177.35 (11)	O15—Co2—N2	177.46 (14)
N1—Co1—O8	92.17 (11)	O14—Co2—N2	87.96 (12)
O4—S1—O5	114.2 (2)	O12—S2—O13	114.3 (2)
O4—S1—O3	112.1 (2)	O12—S2—O11	111.3 (2)
O5—S1—O3	110.4 (2)	O13—S2—O11	110.9 (2)
O4—S1—C9	106.2 (2)	O12—S2—C19	107.3 (2)
O5—S1—C9	107.7 (2)	O13—S2—C19	107.1 (2)
O3—S1—C9	105.58 (17)	O11—S2—C19	105.44 (17)
C1—O1—Co1	130.0 (2)	C11—O9—Co2	129.9 (2)
S1—O3—Co1	131.92 (16)	S2—O11—Co2	132.74 (17)
Co1—O6—H6OA	119 (4)	Co2—O14—H14B	117 (3)
Co1—O6—H6OB	126 (4)	Co2—O14—H14A	122 (3)
H6OA—O6—H6OB	107 (5)	H14B—O14—H14A	109 (4)
Co1—O7—H7OA	138 (4)	Co2—O15—H15A	126 (6)
Co1—O7—H7OB	115 (5)	Co2—O15—H15B	124 (5)
H7OA—O7—H7OB	104 (6)	H15A—O15—H15B	110 (7)
Co1—O8—H8OA	112 (3)	Co2—O16—H16A	128 (3)
Co1—O8—H8OB	112 (3)	Co2—O16—H16B	127 (4)
H8OA—O8—H8OB	112 (5)	H16A—O16—H16B	105 (5)
C7—N1—C8	116.0 (3)	C17—N2—C18	116.3 (3)
C7—N1—Co1	124.5 (2)	C17—N2—Co2	124.2 (2)
C8—N1—Co1	119.3 (2)	C18—N2—Co2	119.4 (2)
O1—C1—C6	123.1 (3)	O9—C11—C16	123.4 (3)
O1—C1—C2	119.5 (3)	O9—C11—C12	119.5 (3)
C6—C1—C2	117.3 (3)	C16—C11—C12	117.0 (3)
C3—C2—C1	122.1 (3)	C13—C12—C11	122.1 (3)
C3—C2—H2	118.9	C13—C12—H12	118.9
C1—C2—H2	118.9	C11—C12—H12	118.9
C2—C3—C4	120.6 (3)	C12—C13—C14	120.8 (3)
C2—C3—H3	119.7	C12—C13—H13	119.6
C4—C3—H3	119.7	C14—C13—H13	119.6
C5—C4—C3	118.1 (3)	C15—C14—C13	118.4 (3)
C5—C4—C10	119.9 (3)	C15—C14—C20	119.5 (3)
C3—C4—C10	121.9 (3)	C13—C14—C20	122.1 (3)
C4—C5—C6	122.9 (3)	C14—C15—C16	122.2 (3)
C4—C5—H5	118.5	C14—C15—H15	118.9
C6—C5—H5	118.5	C16—C15—H15	118.9
C5—C6—C1	118.7 (3)	C15—C16—C11	119.2 (3)
C5—C6—C7	116.4 (3)	C15—C16—C17	116.3 (3)
C1—C6—C7	124.9 (3)	C11—C16—C17	124.5 (3)
N1—C7—C6	127.6 (3)	N2—C17—C16	128.0 (3)
N1—C7—H7	116.2	N2—C17—H17	116.0
C6—C7—H7	116.2	C16—C17—H17	116.0
N1—C8—C9	112.5 (3)	N2—C18—C19	112.6 (3)
N1—C8—H8A	109.1	N2—C18—H18A	109.1
C9—C8—H8A	109.1	C19—C18—H18A	109.1
N1—C8—H8B	109.1	N2—C18—H18B	109.1
C9—C8—H8B	109.1	C19—C18—H18B	109.1
H8A—C8—H8B	107.8	H18A—C18—H18B	107.8
C8—C9—S1	112.6 (3)	C18—C19—S2	112.4 (3)
C8—C9—H9A	109.1	C18—C19—H19A	109.1
S1—C9—H9A	109.1	S2—C19—H19A	109.1
C8—C9—H9B	109.1	C18—C19—H19B	109.1
S1—C9—H9B	109.1	S2—C19—H19B	109.1
H9A—C9—H9B	107.8	H19A—C19—H19B	107.9
O2—C10—C4	125.1 (4)	O10—C20—C14	125.8 (4)
O2—C10—H10	117.5	O10—C20—H20	117.1
C4—C10—H10	117.5	C14—C20—H20	117.1
			
O3—Co1—O1—C1	−111.7 (7)	O11—Co2—O9—C11	113.8 (9)
O7—Co1—O1—C1	−176.3 (3)	O16—Co2—O9—C11	86.0 (3)
O6—Co1—O1—C1	94.2 (3)	O15—Co2—O9—C11	176.1 (3)
N1—Co1—O1—C1	7.6 (3)	O14—Co2—O9—C11	−94.4 (3)
O8—Co1—O1—C1	−84.6 (3)	N2—Co2—O9—C11	−6.5 (3)
O4—S1—O3—Co1	−139.7 (2)	O12—S2—O11—Co2	134.6 (3)
O5—S1—O3—Co1	91.8 (3)	O13—S2—O11—Co2	−97.1 (3)
C9—S1—O3—Co1	−24.4 (3)	C19—S2—O11—Co2	18.5 (3)
O1—Co1—O3—S1	121.5 (7)	O9—Co2—O11—S2	−115.9 (9)
O7—Co1—O3—S1	−173.6 (3)	O16—Co2—O11—S2	−88.1 (3)
O6—Co1—O3—S1	−84.4 (2)	O15—Co2—O11—S2	−178.4 (3)
N1—Co1—O3—S1	2.4 (2)	O14—Co2—O11—S2	92.2 (3)
O8—Co1—O3—S1	94.3 (2)	N2—Co2—O11—S2	4.2 (3)
O1—Co1—N1—C7	0.2 (3)	O9—Co2—N2—C17	−0.8 (3)
O3—Co1—N1—C7	173.8 (3)	O11—Co2—N2—C17	−175.8 (3)
O7—Co1—N1—C7	−95.6 (18)	O16—Co2—N2—C17	−88.0 (3)
O6—Co1—N1—C7	−94.5 (3)	O15—Co2—N2—C17	90 (3)
O8—Co1—N1—C7	87.9 (3)	O14—Co2—N2—C17	93.8 (3)
O1—Co1—N1—C8	174.7 (3)	O9—Co2—N2—C18	−176.8 (3)
O3—Co1—N1—C8	−11.7 (3)	O11—Co2—N2—C18	8.2 (3)
O7—Co1—N1—C8	78.9 (18)	O16—Co2—N2—C18	95.9 (3)
O6—Co1—N1—C8	80.1 (3)	O15—Co2—N2—C18	−86 (3)
O8—Co1—N1—C8	−97.5 (3)	O14—Co2—N2—C18	−82.2 (3)
Co1—O1—C1—C6	−10.9 (5)	Co2—O9—C11—C16	10.3 (5)
Co1—O1—C1—C2	170.5 (2)	Co2—O9—C11—C12	−170.5 (2)
O1—C1—C2—C3	174.6 (3)	O9—C11—C12—C13	−174.2 (3)
C6—C1—C2—C3	−4.1 (5)	C16—C11—C12—C13	5.0 (5)
C1—C2—C3—C4	−0.3 (6)	C11—C12—C13—C14	−0.2 (6)
C2—C3—C4—C5	3.3 (5)	C12—C13—C14—C15	−3.6 (5)
C2—C3—C4—C10	−175.2 (3)	C12—C13—C14—C20	175.1 (3)
C3—C4—C5—C6	−1.9 (5)	C13—C14—C15—C16	2.6 (5)
C10—C4—C5—C6	176.6 (3)	C20—C14—C15—C16	−176.2 (3)
C4—C5—C6—C1	−2.5 (5)	C14—C15—C16—C11	2.3 (5)
C4—C5—C6—C7	179.0 (3)	C14—C15—C16—C17	−178.4 (3)
O1—C1—C6—C5	−173.3 (3)	O9—C11—C16—C15	173.3 (3)
C2—C1—C6—C5	5.3 (5)	C12—C11—C16—C15	−5.9 (5)
O1—C1—C6—C7	5.1 (5)	O9—C11—C16—C17	−6.0 (5)
C2—C1—C6—C7	−176.3 (3)	C12—C11—C16—C17	174.8 (3)
C8—N1—C7—C6	−179.4 (3)	C18—N2—C17—C16	−179.5 (4)
Co1—N1—C7—C6	−4.7 (5)	Co2—N2—C17—C16	4.4 (5)
C5—C6—C7—N1	−178.4 (3)	C15—C16—C17—N2	178.9 (3)
C1—C6—C7—N1	3.1 (6)	C11—C16—C17—N2	−1.9 (6)
C7—N1—C8—C9	−135.3 (3)	C17—N2—C18—C19	134.9 (4)
Co1—N1—C8—C9	49.7 (4)	Co2—N2—C18—C19	−48.8 (4)
N1—C8—C9—S1	−80.4 (4)	N2—C18—C19—S2	81.6 (4)
O4—S1—C9—C8	−177.7 (3)	O12—S2—C19—C18	179.6 (3)
O5—S1—C9—C8	−54.9 (3)	O13—S2—C19—C18	56.5 (4)
O3—S1—C9—C8	63.1 (3)	O11—S2—C19—C18	−61.7 (4)
C5—C4—C10—O2	179.9 (3)	C15—C14—C20—O10	−179.3 (3)
C3—C4—C10—O2	−1.7 (6)	C13—C14—C20—O10	2.0 (6)

### Hydrogen-bond geometry (Å, °)

**Table:**

*D*—H···*A*	*D*—H	H···*A*	*D*···*A*	*D*—H···*A*
O6—H6OA···O2^i^	0.83 (1)	1.95 (1)	2.772 (4)	171 (5)
O6—H6OB···O9^ii^	0.82 (1)	1.95 (2)	2.739 (3)	163 (5)
O7—H7OA···O13	0.82 (1)	2.45 (3)	3.194 (6)	153 (5)
O7—H7OB···O20	0.82 (1)	2.12 (3)	2.877 (9)	155 (7)
O8—H8OA···O17	0.83 (1)	1.92 (1)	2.742 (5)	170 (4)
O8—H8OB···O5^iii^	0.82 (1)	2.00 (1)	2.807 (4)	173 (5)
O14—H14A···O1	0.82 (1)	1.94 (2)	2.732 (3)	163 (5)
O14—H14B···O10^iv^	0.82 (1)	1.97 (1)	2.784 (4)	175 (5)
O15—H15B···O5^iii^	0.82 (1)	2.28 (4)	3.012 (5)	150 (7)
O15—H15A···O19	0.82 (1)	2.17 (5)	2.831 (7)	138 (7)
O16—H16A···O18	0.82 (1)	1.88 (1)	2.691 (5)	175 (4)
O16—H16B···O13^iii^	0.82 (1)	1.95 (2)	2.748 (5)	167 (6)

Symmetry codes: (i) −*x*+1, −*y*, −*z*+1; (ii) *x*−1, *y*, *z*; (iii) *x*+1, *y*, *z*; (iv) −*x*+2, −*y*+1, −*z*+1.
